# Appraisal and patient-reported outcomes following total hip arthroplasty: a longitudinal cohort study

**DOI:** 10.1186/s41687-022-00498-z

**Published:** 2022-09-05

**Authors:** Carolyn E. Schwartz, Bruce D. Rapkin, Jhase Sniderman, Joel A. Finkelstein

**Affiliations:** 1 DeltaQuest Foundation, Inc., 31 Mitchell Road, Concord, MA 01742 USA; 2 Departments of Medicine and Orthopaedic Surgery, Tufts University School of Medicine, Boston, MA USA; 3 Department of Epidemiology and Population Health, Albert Einstein College of Medicine, Bronx, NY USA; 4 Division of Orthopaedic Surgery, University of Toronto, Toronto, ON Canada; 5 Division of Orthopedic Surgery, Sunnybrook Health Sciences Centre, Toronto, ON Canada; 6 Division of Spine Surgery, Sunnybrook Health Sciences Centre, Toronto, ON Canada

**Keywords:** Total hip arthroplasty, Quality of life, Physical functioning, Mental-health functioning, Cognitive appraisal

## Abstract

**Background:**

Total hip arthroplasty (THA) is a successful procedure that provides pain relief, restores function, and improves quality of life (QOL) for patients with advanced arthritis in their hip joint. To date, little research has examined the role of cognitive appraisal processes in THA outcomes. This study examined the role of cognitive appraisal processes in THA outcomes in the first year post-surgery.

**Methods:**

This longitudinal cohort study collected data at pre-surgery, 6 weeks post-surgery, 3 months post-surgery, and 12 months post-surgery. Adults (n = 189) with a primary diagnosis of osteoarthritis were consecutively recruited from an active THA practice at a Canadian academic teaching hospital. Measures included the Hip Disability and Osteoarthritis Outcome Score (HOOS), the Mental Component Score (MCS) of the Rand-36, and the Brief Appraisal Inventory (BAI). Analysis of Variance examined the association between BAI items and the HOOS or MCS scores. Random effects models investigated appraisal main effects and appraisal-by-time interactions for selected BAI items.

**Results:**

HOOS showed great improvement over the first 12 months after THA, and was mitigated by three appraisal processes in particular: focusing on problems with healthcare or living situation, and preparing one’s family for health changes. MCS was stable and low over time, and the following appraisal processes were implicated by very large effect sizes: not comparing themselves to healthier people, focusing on money problems, preparing their family for their health changes, or trying to shed responsibilities.

**Conclusions:**

Appraisal processes are relevant to health outcomes after THA, with different processes coming into play at different points in the recovery trajectory.

**Supplementary Information:**

The online version contains supplementary material available at 10.1186/s41687-022-00498-z.

## Introduction

Total hip arthroplasty (THA) is a successful procedure that provides pain relief, restores function, and improves quality of life (QOL) for patients with advanced arthritis in their hip joint [1–3]. Measures of physical functioning show very positive outcomes of THA [4, 5]. The recovery trajectory for THA is rapid for physical impairment (by 2 weeks), and almost all patients report improved mood by 1–2 months. By 3 months post-surgery, a very high proportion (87–93%) of patients report full recovery in physical functioning and activities of daily living [6, 7], and are satisfied with the surgical outcome [3, 8–11].

Despite this high success rate, a substantial body of research has focused on identifying clinical and psychosocial predictors of poor outcomes [3, 12, 13]. Preoperative mental health scores have been found to predict functional outcomes at one-year post-surgery [14]. Despite a successful surgery, mental health does not change substantially over the year after surgery [15]. Thirty-seven percent of patients suffer from clinical depression preoperatively and continue to do so at one year post-surgery [16]. This is a higher proportion than in the general population (24%) of people age 65 and older [17].

More resilient patients have been found to report higher satisfaction and better health status post-surgery [18], and have more socioeconomic resources [13, 19, 20]. In addition to such external resources, research in other therapeutic areas has documented the importance of internal cognitive resources, specifically how one thinks about health and quality of life (QOL) [21, 22]. For example, cognitive appraisals focused on more positive and controllable aspects of one’s condition are associated with better physical and mental-health outcomes [23]. Further, focusing more on one’s personal goals has been found to be associated with better recovery trajectories after orthopaedic surgery [24].The influence of stressors like injury or financial difficulties on evaluative ratings of pain or financial distress depends on the ways that people understand and think about their QOL [23, 25–32]. In a recent analysis of factors that help predict postoperative outcomes following THA, patients' cognitive appraisal processes were critical in determining their functional outcome [33].

Detecting and accounting for such inter- and intra-individual differences in appraisal can be important for understanding the impact of THA on health outcomes over the recovery trajectory. Changes in appraisal processes over time can lead to response-shift effects, if these changes explain variance in the discrepancy between expected and observed QOL [21, 34]. Appraisal measures are *idiometric* in that they assess thought processes that are contingent on circumstances, and thus do not reduce to simple scale scores that are consistent across samples [35]. Consequently, one must examine appraisal processes individually (i.e., as separate items) [35]. As a main effect, appraisal can highlight underlying differences in how people think about QOL that impact or obfuscate score differences between groups [29]. As a time-varying effect, appraisal changes over time may reflect adaptation to changing health [21]. Appraisal assessment can help to portray individual differences in terms that depict how QOL concerns and priorities influence their evaluation of physical and mental health.

This study examined the role of cognitive appraisal processes in THA outcomes in the first year post-surgery. We focused on one outcome with documented responsiveness to THA (i.e., physical functioning) [4, 5] and one that seems to yield relatively small effects after THA (i.e., mental-health functioning) [15]. We hypothesized that appraisal processes would help to explain differences over time in the responsive outcome, and that underlying changes in appraisal processes would clarify lack of change on the non-responsive outcome.

## Methods

### Sample and design

This prospective longitudinal cohort study included adults who were consecutively recruited from an active total hip arthroplasty (THA) practice at a Canadian academic teaching hospital. Eligibility criteria included being over the age of 18 and having a primary diagnosis of osteoarthritis. Patients were excluded if they had diagnoses of osteonecrosis, cancer, acute fracture, or inflammatory arthritis; were on immunosuppressant medications; had undergone a previous total joint arthroplasty; did not provide informed consent; or were unable to understand and complete the English survey-related documents. All patients provided written informed consent prior to completing any questionnaires. Data were collected online or by mail pre-surgery and again at roughly 6 weeks post-surgery, 3 months post-surgery, and 12 months post-surgery using a secure, Health Information Portability and Accountability Act (HIPAA)-compliant interface [36]. The study was reviewed and approved by the institution’s Research Ethics Board (Sunnybrook Health Sciences Research Ethics Board protocol 296-2008).

### Measures

The Hip Disability and Osteoarthritis Outcome Score (HOOS) [37] assessed physical functioning. This 19-item measure assesses pain (2 items) and degree of difficulty engaging in activities of daily living (17 items). The summary score was computed as the mean of the individual items, divided by 4 (the highest possible score for a single answer option), multiplied by 100 as a scaling factor, and then subtracted from 100 so that higher scores would reflect better outcomes.

The Mental Component Score (MCS) of the Medical Outcomes Study Short-Form (Rand-36) [38–40] assessed mental-health functioning. This score is created by summing the eight standardized domain scores weighted by factor score coefficients that lend the most weight to the mental health, role emotional, social functioning, and vitality domains [41, 42]. The scores are then transformed to a norm-based T-score, with a mean of 50 and standard deviation of 10 [42]. The population norm is a score of 50 [41]. Higher scores reflect better outcomes.

Cognitive appraisal processes were measured by the Brief Appraisal Inventory (BAI) [43]. The BAI is an idiometric measure, meaning it assesses context- and circumstance-specific patient experience and meaning [35]. While data reduction techniques like principal components analysis have been used in some past research with the BAI (e.g., [44, 45]), the component structure varies across participant population [35]. This variability is to be expected for an idiometric measure [35], but also makes it difficult to compare results across studies. Accordingly, to facilitate comparison across studies, the BAI and other measures of appraisal are now more commonly analyzed at the item-level [33, 35, 46]. Each of the 22^1^BAI items encompasses overarching patterns of appraisal gleaned from a series of studies in medically ill patient groups [47, 48].^2^ Items utilized a 5-point rating scale (Never, Rarely, Sometimes, Often, Always), with higher values assigned to more endorsement.

To describe the sample, clinical and demographic variables were collected. Exercise practice, which has demonstrated relevance to post-orthopaedic-surgery outcomes [49], was assessed using the DeltaQuest Reserve-Building Activities Measure© Exercise subscale, which tracks the number of days per week of mild (minimal effort, such as easy walking, easy yoga), moderate (not exhausting, such as fast walking, easy cycling, easy swimming), and strenuous exercise (heart beats rapidly; such as running, vigorous swimming, vigorous long-distance bicycling) [50].

### Statistical analysis

Descriptive statistics were used to summarize the study sample. Univariate Analysis of Variance (ANOVA) models examined the association between BAI items (independent variables) and HOOS or MCS (dependent variables in separate models). We tested each time window specified in the study protocol separately: pre-surgery (within 200 days^3^), 6 weeks (± 1 week), 3-months (± 2 weeks) and 12-months (± 6 weeks). Appraisal was also treated as a categorical variable. This treatment enables detection of non-linear relationships and does not presume equal intervals across response options.

To guide interpretation of patterns in explained variance over study time windows, Cohen’s published cut-offs for explained variance (eta^2^) were used [51]. We then computed random effects models (REM) [52] to test appraisal main effects and appraisal-by-time interactions for those BAI items that met the above criterion.

*Adjusting for Multiple Comparisons* To reduce the risk of false rejection of the null hypothesis, we adopted a family-wise comparison rate of α = 0.05, where the “family” was defined as the 22 appraisal comparisons for each outcome. An eta^2^ of 0.206 met this criterion. This is greater than what Cohen would classify as a large effect (eta^2^ of 0.14) [53]. REMs were only run for those items that met this criterion.

*Observation Selection* These data are being collected in the context of an active orthopaedic clinic, with variable amounts of follow-up by patient. Accordingly, data are not missing but rather not yet collected. The hypothesis-driven data analysis was accomplished by filtering observations from the larger data set (n = 790 observations) to include people with at least one observation before and at least one after surgery, and who had data on the HOOS, MCS and BAI. Observations were further filtered to include those that fit into the time windows specified above.

*Software* Data were analyzed using IBM SPSS version 27 [54] and the R software [55].

## Results

### Sample

The study sample included 189 people who underwent THA between July 2018 and June 2021. About half of the patients received a direct lateral approach, with the other half divided between anterolateral (25%) and direct anterior Arthroplasty approaches (22%). Table 1 provides descriptive statistics on the sample. Patients contributed an average of 2.6 observations (visits), and the average follow-up was 308 days or about ten months (SD = 146 days or about 5 months; Table 1).

**Table 1:** Descriptive statistics of study sample at baseline† (N = 189)

Variable	Mean	SD
Age	66.5	9.5
Range	34–85	
Comorbidities*, out of 14 presented	1.1	1.5
Range	0–6	
BMI	28.5	5.1
Range	11.8–40.0	
HOOS summary score (higher is better)	69.8	24.1
Range	0–100	
MCS (SF36 mental component) (higher is better)	44.9	8.1
Range	12.1–60.9	
Follow-up time in days	308	146
Range	34–805	
Exercise (> 15 min; number of days per week, range 0–7)		
Strenuous (heart beats rapidly)	1.2	1.8
Moderate (not exhausting)	3.1	2.4
Mild (minimal effort)	4.6	2.4

	Frequency	**%**
Surgical approach		
Anterolateral	47	25
Direct Lateral	96	51
Direct Anterior Arthroplasty	41	22
Missing	5	3
Specific comorbidities*		
Back pain	98	52
Arthritis	173	92
Asthma	13	7
Cancer—now or in the past	22	12
Depression	29	15
Diabetes	12	6
Heart disease	16	8
High blood pressure	63	33
Insomnia	36	19
Kidney disease	4	2
Liver disease	0	0
Lung disease	3	2
Stroke	4	2
Ulcer or stomach disease	6	3
Other	41	22
Smoking status		
Never Smoked	98	52
Used to Smoke	75	40
Currently Smoke	13	7
Missing	3	2
Gender		
Male	92	49
Female	97	51
Marital status		
Married	124	66
Widowed	13	7
Living with significant other	11	6
Single (never married)	15	8
Divorced/Separated	22	12
Civil Union/Domestic Partner	4	2
Race		
First Nations/Native Canadian	2	1
Asian	5	3
Black	2	1
White	172	91
Missing	8	4
Hispanic ethnicity		
Yes	2	1
No	102	54
Missing	85	45
Work status		
Currently working	68	36
On leave of absence	5	3
Retired (not due to ill health)	95	50
Disabled and/or retired because of ill health	4	2
Homemaker	1	1
Unemployed	1	1
Other	15	8
Hours worked per week		
Does not apply	93	49
Less than 20 h	25	13
20–29 h	8	4
30–39 h	17	9
40 or more hours	42	22
Missing	4	2
Workers' compensation for current condition (on or planning to apply)		
Yes	1	1
No	180	95
Missing	8	4
Considering legal action for current condition		
Yes	2	1
No	177	94
Missing	10	5
Level of education		
Less than high school	8	4
Graduated from high school or earned GED	16	8
Some college or technical school	29	15
Graduated from college	53	28
Postgraduate school or degree	81	43
Missing	2	1

†Data reflect baseline values for all variables except Follow-up Time

*For these topics, a non-response was counted as the absence of the condition in question

### Change in outcomes over time

**Fig. 1:**
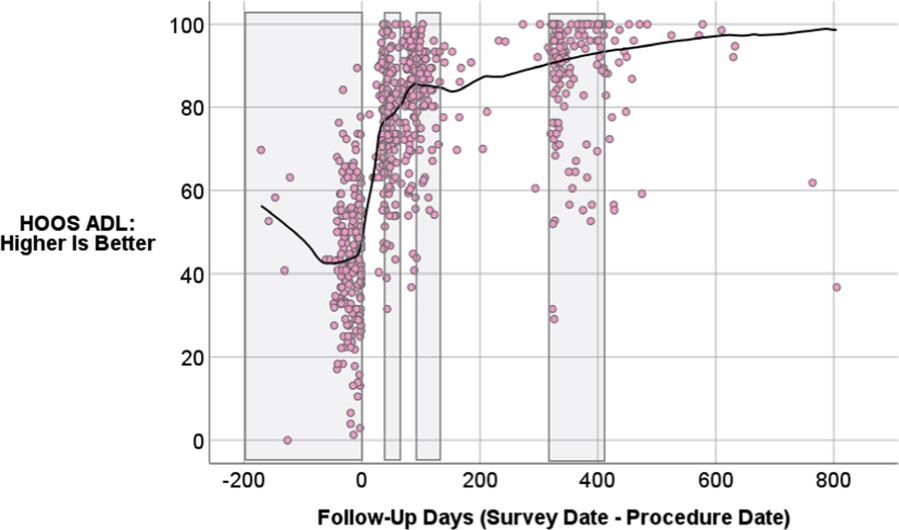
Scatter plot of HOOS scores over time

**Fig. 2:**
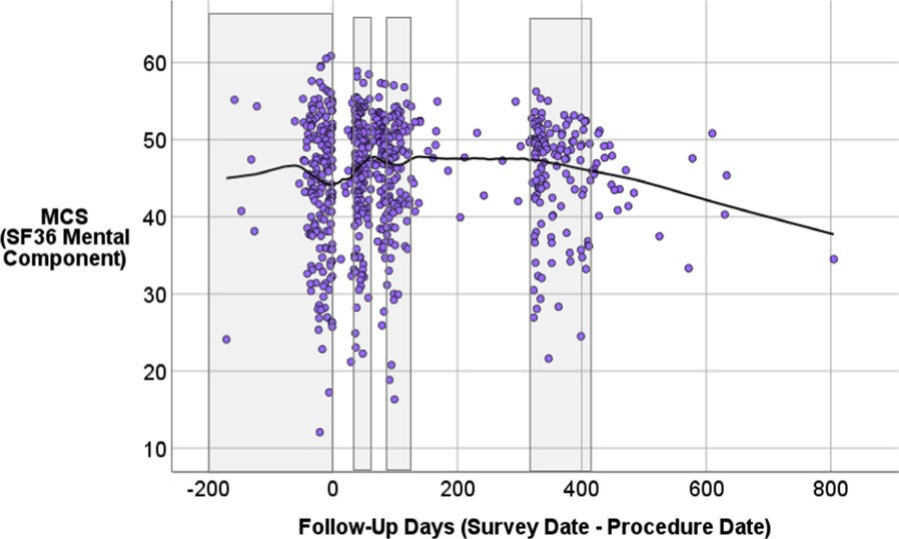
Scatter plot of MCS scores over time

Figures 1 and 2 show scatter plots of HOOS and MCS scores over time. Locally Weighted Scatterplot Smoothing (lowess) lines show the trends over time on these two outcome variables. These figures show the full range of data collected to date so that we could assess whether the lowess looks different after 12 months or appears to continue in the same trajectory. We did not have adequate sample sizes to include longer-term follow-up in the formal models. The HOOS appears to be responsive to THA surgery at the aggregate level, while the MCS seems only minimally responsive to surgery. Additionally, the sample was lower than the population norm on this mental-health functioning score, which is about 52 in a similar age cohort [41].

### Association of appraisal with HOOS physical functioning

Table 2 provides the eta^2^ estimates for HOOS predicted by BAI items at each time window. Conditional formatting shows the ES magnitude using Cohen’s criteria as cut-offs [51]. Appraisal processes explain less variance and are thus less associated with physical functioning at pre-surgery than at subsequent time points.

**Table 2:**
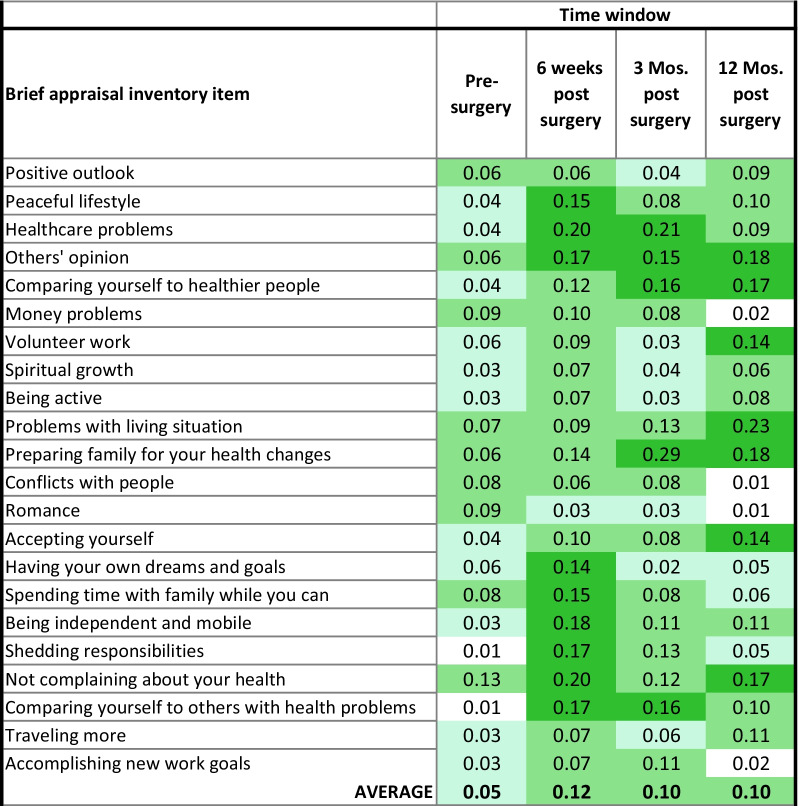
HOOS: Eta^2^ in one-way ANOVA

Conditional formatting reflects effect-size magnitude per Cohen. Light blue shading reflects small ES, light green reflects medium ES, and dark green reflects large ES

*Threshold for significance of each ANOVA eta2 was set at 0.206, maintaining a familywise alpha at approximately 0.01

There were three general patterns of association between appraisal and physical functioning over time (Table 2). Some items explained more variance *early in the recovery trajectory* (i.e., at 6 weeks or at 6 weeks and 3 months; e.g., peaceful lifestyle, focusing on healthcare problems). Other appraisal items explained more variance across the recovery trajectory, and this association was *relatively constant* (e.g., focusing on other’s opinion, comparing self to healthier people,). Still other appraisal items became more important (i.e., explained more variance) *later in the recovery trajectory* (e.g., focusing on problems with living situation, accepting yourself).

**Fig. 3:**
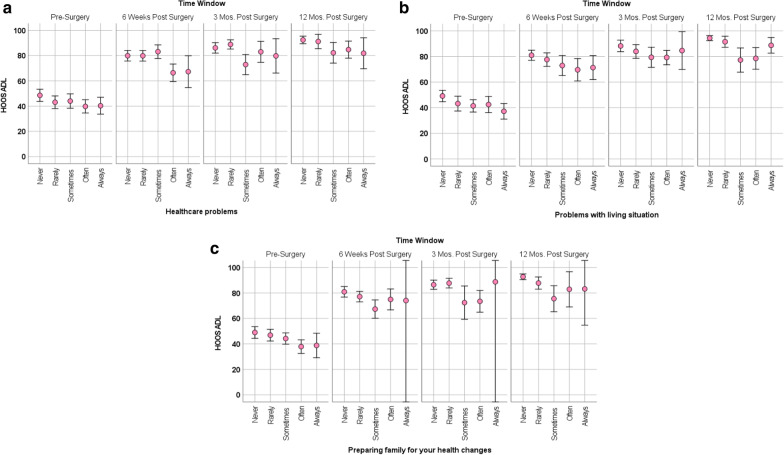
**a**–**c** Main effects of appraisal with HOOS over time

Additional file 1: Supplemental Table 1 provides results of the REMs evaluating the main effects of time, the three appraisal items with the largest eta^2^ estimates, and appraisal-by-time interactions. It is notable that HOOS demonstrated significant longitudinal change (F_time window_ = 390.25, *p* < 0.001). These models support main effects of appraisal constituted by focusing on healthcare problems, problems with living situation, and preparing family; but do not support any appraisal-by-time interactions. Thus, those who focused less on appraisal processes related to these practical demands of life reported better physical functioning at all points in time (Additional file 1: Supplemental Table 1; Fig. 3a–c).

### Association of appraisal with MCS mental-health functioning

In contrast to physical functioning, mental-health functioning is associated more strongly with appraisal pre-surgically (average eta^2^ was 0.10 for MCS at pre-surgery as compared to 0.05 for HOOS; Table 3). Specifically, focusing on others’ opinions and on not complaining about one’s health are large-ES predictors, and most others are medium-ES predictors.

**Table 3:**
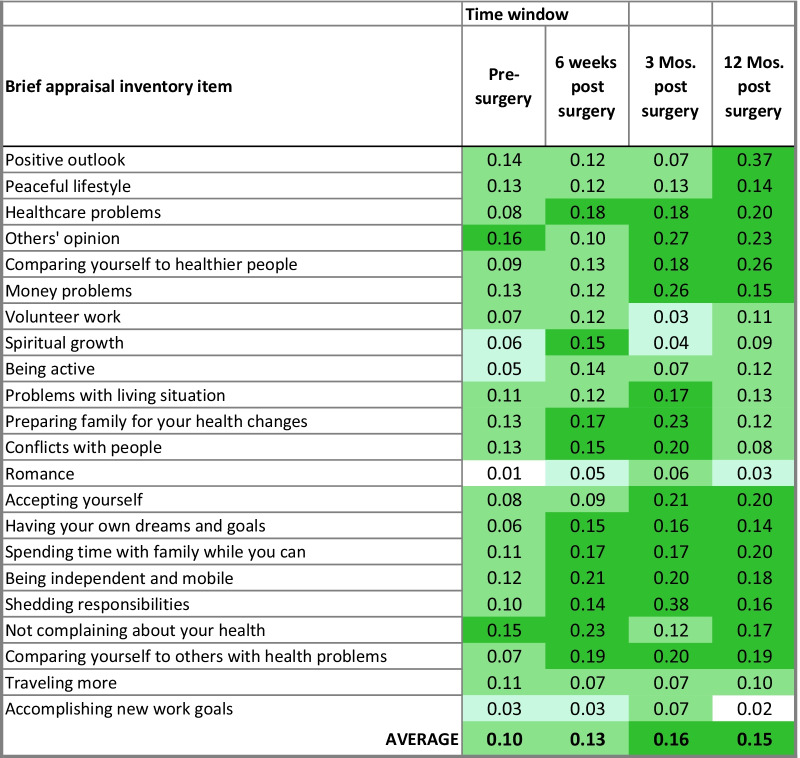
MCS: Eta^2^ in one-way ANOVA

Conditional formatting reflects effect-size magnitude per Cohen. Light blue shading reflects small ES, light green reflects medium ES, and dark green reflects large ES

*Threshold for significance of each ANOVA eta2 was set at 0.206, maintaining a familywise alpha at approximately 0.01

Once surgery has occurred, appraisal items explain more variance with time. About half of the appraisal items are important for mental-health functioning. They have large ES as early as 6 weeks, and often no later than 3 months post-surgery, and stay important throughout the first-year post-surgery (e.g., being independent and mobile, having own dreams and goals). These associations were *relatively constant*. Some appraisal items became more important *later in the recovery trajectory* (e.g., positive outlook, comparing self to healthier people). Others were relatively important but not more at any particular time window (e.g., peaceful lifestyle, traveling more), and other were unimportant throughout (e.g., romance, accomplishing new work goals).

Additional file 1: Supplemental Table 2 provides results of the REMs evaluating the main effect of the eight appraisal items with the largest eta^2^ estimates in predicting MCS scores over time and appraisal-by-time interactions. It is notable that MCS did not demonstrate significant longitudinal change at the aggregate level (F_time window_ = 1.79, *p* = 0.15). Rather, main effects in the MCS models demonstrate multiple strong relationships between mental-health functioning and appraisal.

**Fig. 4:**
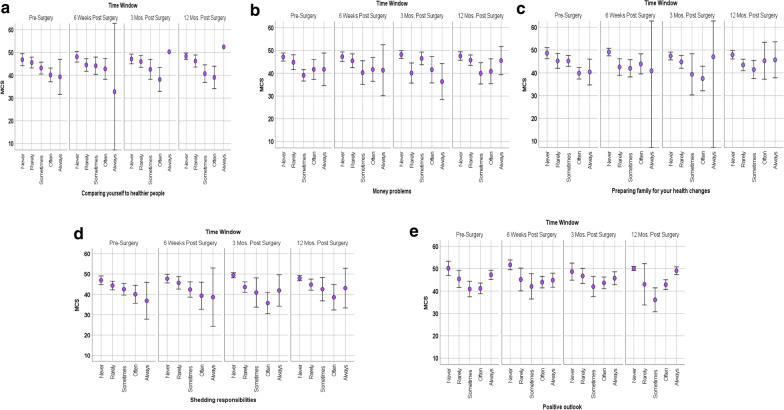
**a**–**e** Main effects of appraisal with MCS over time

Specifically, based on large eta^2^ ranging from 0.23 to 0.38, mental health was most strongly associated with individual differences in comparing oneself to healthier people,^4^ money problems, preparing family, shedding responsibilities, and maintaining a positive outlook. Those who endorsed less frequent comparison to the first three items shown in Fig. 4a–c reported better mental-health functioning. In contrast, focusing on having a positive outlook seldom or always yielded better mental health. This u-shaped curve is especially evident in Fig. 4e at pre-surgery and 12 months post-surgery. While other plots may look slightly u-shaped, they are limited by having very few points in the response option that suggests a u-shape. We believe only Fig. 4e illustrates a robust u-shaped relationship in these data.

**Fig. 5:**
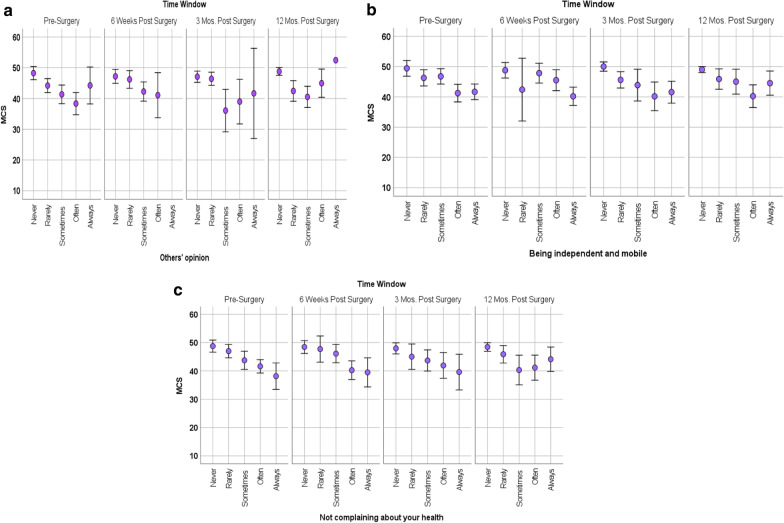
**a**–**c** Interactions of appraisal with MCS over time

There were also significant appraisal-by-time interactions for focusing on others’ opinion, being independent, and not complaining about one’s health (Additional file 1: Supplemental Table 2; Fig. 5a–c). Focusing less often on these three appraisal processes was associated with better mental health. Over time (i.e., at all timepoints), the people more focused on those concerns showed the greatest improvement in MCS.

## Discussion

The present study revealed that appraisal processes are relevant to health outcomes after THA, with different processes coming into play at different points in the recovery trajectory. While the HOOS showed great improvement over the first 12 months after THA (i.e., a responsive outcome), it was mitigated by three appraisal processes in particular. Focusing more on problems with healthcare or living situation, and preparing one’s family for health changes were generally associated with worse HOOS scores post-surgery.

Whereas time since surgery accounted for substantial variance in the HOOS, time was not strongly related to the MCS (i.e., a non-responsive outcome). Accordingly, there is more individual variation that could be explained by individual differences in appraisal. Indeed, certain appraisal processes were implicated in maintaining this apparently stable albeit low mental-health functioning. Specifically, those who reported better mental-health functioning tended to be those who did *not* compare themselves to healthier people, focus on money problems, prepare their family for their health changes, or try to shed responsibilities. It is noteworthy that the significant MCS-by-time interactions all concerned appraisals related to not wanting to be a burden on their family: being independent, not complaining, and being concerned with others’ impressions or opinions. Those who did *not* focus on these interpersonal concerns reported better mental health.

In addition to these more linear relationships, we found a non-linear relationship with one appraisal item in particular. People who reported better mental-health functioning generally tended to never/rarely *or* often/always focus on having a positive outlook. This u-shaped pattern may reflect individual differences in coping: For people on one extreme (never, rarely), maintaining a positive outlook is not an issue. For people at the other extreme (often, always), they have found a way to be effective at such. For people in the middle, however, their answer of “sometimes” may represent their struggle to find a way to cope. In our context, patients who persisted in a particular cognitive approach—either not engaging or regularly engaging in trying to be positive—reported better mental-health functioning.

### Clinical implications

The clinical implications of this work can be applied to already established perioperative pathways. Enhanced Recovery After Surgery (ERAS) is a multimodal approach that is increasingly used in the care of surgical patients [56]. ERAS pathways for total joint arthroplasty lower costs, mortality and length of stay, and improve patient satisfaction [57], but fail to address many of the social and psychological aspects that are important to patients undergoing surgery. Building on the recent work showing that patient’s cognitive appraisal processes were critical in determining their functional outcome following THA [33], as well as the present work, preoperative identification of at-risk patients would be the first step in addressing this. The BAI could be used as a screening tool. Patients identified as having significant concerns about their living situation, family involvement, employment and health changes following surgery could be optimized with a “prehabilitation” type approach [58–61]. As part of this prehabilitation, clinicians from a range of disciplines (e.g., physicians, nurses, physical and occupational therapists, social workers, and counselors) could work together to prepare patients for surgery.

For example, appraisals related to preparing one’s family for one’s health changes explained substantial variance in both HOOS and MCS scores over time, and particularly at 3- and 6-months post-surgery for the HOOS, and at 6-weeks and 3-months post-surgery for the MCS. Those who focused more on such appraisals generally reported worse outcomes. A prehabilitation approach might involve talking with patients about how such a focus is not adaptive, and that a better approach would be to focus on improving their living situation and on activities that help them maintain a positive outlook (related to improved HOOS and MCS, respectively in the present work). Since this association may also reflect on the patient’s family situation, prehabilitation might also include conferences and education for caregivers, to help them better understand what to expect following surgery.

### Limitations

The present work has the advantage of a longitudinal design with data collected at clinically relevant milestones with regard to THA recovery. Its limitations must, however, be acknowledged. First, the BAI was analyzed at the item level rather using data-reduction techniques. This is consistent with standards for analysis of idiometric measures [35]. This led to a number of statistical comparisons which could have inflated the Type I error rate. Second, treating appraisal responses as categorical rather than continuous also increased the size of the eta^2^. In response to both of these concerns, we corrected for such by allowing a family-wise Type I error rate of 0.01 for each patient-reported outcome. Such a strict correction may have led to higher Type II error, i.e., missing or ignoring clinically important relationships. Additionally, the sample was relatively small, and thus was likely underpowered for interaction analyses (in REM) or for adjusting for other covariates. Further, the follow-up was relatively short. Findings may differ with longer follow-up. Finally, the sample was predominantly White and educated, thus limiting our study’s generalizability to other race/ethnicity groups and people with less education and other resources [13]. Future work is needed to replicate the analyses in a larger, more diverse sample.

## Conclusions

In summary, fewer appraisal processes explained substantial variance in the responsive outcome of physical functioning than in the non-responsive outcome of mental-health functioning. Thus, consistent with our hypothesis, changes in appraisal processes may clarify lack of change on this non-responsive outcome. Appraisals focused on difficult life challenges were associated with worse outcomes overall, and those focused on being concerned with burdening others were associated with worse mental-health functioning, particularly over time. The clinical implications of this work may involve practical support for life challenges, and emotional support to reframe dependency during recovery so that it is experienced as less worrisome. Clinicians might explicitly discuss with patients the importance of how they think about health during the first year after THA, and the importance of considering contextual demands in coping with the long-term recovery trajectory after THA.

## Supplementary Information

**Additional file 1. Supplemental Table 1.** Random Effects Models Testing Appraisal and Time Effects on HOOS Scores (n=503). **Supplemental Table 2.** Random Effects Models Testing Appraisal and Time Effects on MCS Scores (n=493).

## Data Availability

The study data are confidential and thus not able to be shared.

## Abbreviations

ES: Effect size
HIPAA: Health Information Portability and Accountability Act
HOOS: Hip Disability and Osteoarthritis Outcome Score
MCS: Mental component score
QOL: Quality of life
SD: Standard deviation
THA: Total hip arthroplasty

## Footnotes

1 The original BAI contains 23 items. For the present study, we worked with only 22 items. The item not used refers to “Things that do not usually come to mind, except because of this survey”, which we have found difficult to interpret in this context.

2 Interested readers may contact the first author if they wish to use the measure in their work.

3 In this Canadian clinic, patients are recruited for our study when they first see the hip surgeon to discuss having surgery. They are only referred to the clinic because their condition is likely to benefit from surgery. They are then placed on a list for surgery to be scheduled, and may wait a number of months before actually having the surgery (i.e., a maximum of 200 days). During this time, their symptoms remain the same or worse. This first questionnaire is collected at the time of the first visit and is their baseline questionnaire.

4 It should be noted that “always” often had a large confidence interval, reflecting few observations with that response. For example, the number of patients who “always” compared themselves to healthier people at 6 weeks was 3, at 3 months was 1, and at 12 months was 1. The number of patients who “always” thought about preparing family for their health changes at 6 weeks was 2, and at 3 months was 2.
