# Supplementary material for: Appraisal and patient-reported outcomes following total hip arthroplasty: a longitudinal cohort study
Source: J Patient Rep Outcomes. 2022 Sep 5;6:93. doi: 10.1186/s41687-022-00498-z (PMC9445109; doi:10.1186/s41687-022-00498-z)
Supplement: Supplementary file 1 — Additional file 1. Supplemental Table 1. Random Effects Models Testing Appraisal and Time Effects on HOOS Scores (n=503). Supplemental Table 2. Random Effects Models Testing Appraisal and Time Effects on MCS Scores (n=493). [file 41687_2022_498_MOESM1_ESM.pdf]

Supplemental Table 1. Random Effects Models Testing Appraisal and Time Effects on HOOS Scores (n=503)

Type III Tests of Fixed Effects: Dependent Variable: HOOS Score

Appraisal Item Content	Model	Source	Numerator df	Denominator df	F	Sig.	Akaike's Information Criterion (AIC)	Schwarz's Bayesian Criterion (BIC)
	**Baseline Model for all HOOS models**	Intercept	1	196.39	6771.52	**0.00**	4099.78	4108.21
		Time Window	3	366.97	390.25	**0.00**		
**Healthcare problems**								
	**Appraisal Main-Effect Model**	Intercept	1	208.10	6681.82	**0.00**	4073.15	4081.56
		Time Window	3	366.68	363.55	**0.00**		
		Appraisal: Healthcare problems	4	489.43	3.81	**0.00**		
	**Appraisal-by-time Interaction Model$^{\psi}$**	Intercept	1	213.36	6655.68	**0.00**	3999.96	4008.32
		Time Window	3	367.63	321.45	**0.00**		
		Appraisal: Healthcare problems	4	474.98	4.08	**0.00**		
		Appraisal * Time Window	12	414.56	1.26	0.24		
**Problems with living situation**								
	**Appraisal Main-Effect Model**	Intercept	1	206.88	6923.29	**0.00**	4058.73	4067.14
		Time Window	3	370.68	374.31	**0.00**		
		Appraisal: Problems with living situation	4	483.79	7.66	**0.00**		
	**Appraisal-by-time Interaction Model$^{\psi}$**	Intercept	1	210.70	6744.70	**0.00**	3989.28	2997.64
		Time Window	3	362.53	339.62	**0.00**		
		Appraisal: Problems with living situation	4	470.27	7.63	**0.00**		
		Appraisal * Time Window	12	412.77	0.96	0.48		
**Preparing family for your health changes**								
	**Appraisal Main-Effect Model**	Intercept	1	254.92	5503.67	**0.00**	4056.73	4065.13
		Time Window	3	381.20	291.44	**0.00**		
		Appraisal: Preparing family for health changes	4	490.16	8.10	**0.00**		
	**Appraisal-by-time Interaction Model$^{\psi}$**	Intercept	1	354.55	4215.98	**0.00**	3978.48	3986.84
		Time Window	3	380.54	222.43	**0.00**		
		Appraisal: Preparing family for health changes	4	466.06	8.27	**0.00**		
		Appraisal * Time Window	12	411.11	1.42	0.15		

Estimates of Fixed Effects: Dependent Variable: HOOS Score								
	Parameter		Mean Difference from Pre-Surgery	Std. Error	df	Sig.	95% Confidence Interval	
**Baseline Model**								
	Time Window							
		Pre-surgery	*referent*					
		6 weeks post-surgery	32.47	1.54	361.54	**0.00**	29.45	35.49
		3 mo. post-surgery	40.23	1.62	367.96	**0.00**	37.06	43.41
		12 mo. post-surgery	43.89	1.51	358.98	**0.00**	40.93	46.86
**Healthcare problems**								
**Appraisal Main-Effect Model**	Time Window							
		Pre-surgery	*referent*					
		6 weeks post-surgery	31.91	1.56	357.45	**0.00**	28.84	34.97
		3 mo. post-surgery	39.27	1.65	373.22	**0.00**	36.03	42.50
		12 mo. post-surgery	43.46	1.53	356.91	**0.00**	40.45	46.46
	Appraisal: Healthcare problems							
		Never	*referent*					
		Rarely	-1.37	1.88	494.60	0.46	-5.07	2.32
		Sometimes	-4.87	1.96	492.99	**0.01**	-8.73	-1.01
		Often	-6.10	2.00	475.65	**0.00**	-10.03	-2.17
		Always	-6.81	2.38	494.23	**0.00**	-11.49	-2.13
**Problems with living situation**								
**Appraisal Main-Effect Model**								
	Time Window							
		Pre-surgery	*referent*					
		6 weeks post-surgery	31.70	1.54	361.51	**0.00**	28.66	34.73
		3 mo. post-surgery	39.87	1.62	373.00	**0.00**	36.68	43.06
		12 mo. post-surgery	43.10	1.51	363.30	**0.00**	40.13	46.06
	Appraisal: Problems with living situation							
		Never	*referent*					
		Rarely	-3.80	1.93	490.85	**0.05**	-7.59	-0.01
		Sometimes	-9.02	1.86	491.69	**0.00**	-12.66	-5.37
		Often	-8.16	1.97	489.97	**0.00**	-12.03	-4.30
		Always	-7.30	2.20	487.50	**0.00**	-11.62	-2.99
**Preparing family for your health changes**								
**Appraisal Main-Effect Model**								
	Time Window							
		Pre-surgery	*referent*					
		6 weeks post-surgery	30.27	1.59	377.17	**0.00**	27.15	33.40
		3 mo. post-surgery	37.37	1.69	392.69	**0.00**	34.05	40.69
		12 mo. post-surgery	41.03	1.58	385.44	**0.00**	37.91	44.15
	Appraisal: Preparing family for health changes							
		Never	*referent*					
		Rarely	-1.47	1.72	494.03	0.39	-4.84	1.90
		Sometimes	-7.59	1.89	493.47	**0.00**	-11.31	-3.87
		Often	-9.97	2.08	474.74	**0.00**	-14.06	-5.88
		Always	-8.10	2.98	494.80	**0.01**	-13.94	-2.25

ᵠ Estimates of fixed effects not shown for this model because interaction was not statistically significant. Note: Bolded values have statistically significant p-values ($p<0.05$).

Supplemental Table 2. Random Effects Models Testing Appraisal and Time Effects on MCS Scores (n=493)								
**Type III Tests of Fixed Effects: Dependent Variable: MCS Score**								
Appraisal Item Content	Model	Source	Numerator df	Denominator df	F	Sig.	Akaike's Information Criterion (AIC)	Schwarz's Bayesian Criterion (BIC)
	**Baseline Model for all MCS models**	Intercept	1	200.99	7253.71	**0.00**	3337.92	3346.30
		Time Window	3	340.14	1.79	0.15		
**Positive outlook**								
	**Appraisal Main-Effect Model**	Intercept	1	229.96	7402.45	**0.00**	3299.64	3308.01
		Time Window	3	338.17	1.14	0.33		
		Appraisal: Positive Outlook	4	459.23	8.28	**0.00**		
	**Appraisal-by-time Interaction Model**$^{\psi}$	Intercept	1	245.41	7116.07	**0.00**	3244.48	3252.80
		Time Window	3	335.64	0.54	0.66		
		Appraisal: Positive Outlook	4	437.86	7.25	**0.00**		
		Appraisal * Time Window	12	356.98	1.20	0.28		
**Others' opinion**								
	**Appraisal Main-Effect Model**	Intercept	1	318.95	5271.45	**0.00**	3294.14	3302.51
		Time Window	3	342.14	0.53	0.66		
		Appraisal: Others' opinion	4	448.86	9.69	**0.00**		
	**Appraisal-by-time Interaction Model**	Intercept	1	404.60	4572.33	**0.00**	3231.29	3239.61
		Time Window	3	339.75	3.55	**0.01**		
		Appraisal: Others' opinion	4	412.00	10.25	**0.00**		
		Appraisal * Time Window	11	362.37	2.22	**0.01**		
**Comparing yourself to healthier people**								
	**Appraisal Main-Effect Model**	Intercept	1	315.64	5339.85	**0.00**	3306.10	3314.47
		Time Window	3	338.69	0.63	0.59		
		Appraisal: Comparing yourself to healthier people	4	432.17	6.52	**0.00**		
	**Appraisal-by-time Interaction Model**$^{\psi}$	Intercept	1	412.02	4296.50	**0.00**	3241.82	3250.14
		Time Window	3	330.27	3.19	**0.02**		
		Appraisal: Comparing yourself to healthier people	4	390.20	6.30	**0.00**		
		Appraisal * Time Window	12	354.73	1.71	0.06		

**Money problems**							
**Appraisal Main-Effect Model**	Intercept	1	237.49	6201.81	**0.00**	3308.42	3316.79
	Time Window	3	337.41	1.14	0.33		
	Appraisal: Money problems	4	462.25	5.91	**0.00**		
**Appraisal-by-time Interaction Model**[ψ]	Intercept	1	247.71	6048.84	**0.00**	3247.06	3255.37
	Time Window	3	337.34	1.78	0.15		
	Appraisal: Money problems	4	445.04	4.82	**0.00**		
	Appraisal * Time Window	12	360.04	1.62	0.08		
**Preparing family for your health changes**							
**Appraisal Main-Effect Model**	Intercept	1	255.32	6324.71	**0.00**	3306.00	3314.37
	Time Window	3	347.75	0.01	1.00		
	Appraisal: Preparing family for your health changes	4	444.47	6.66	**0.00**		
**Appraisal-by-time Interaction Model**[ψ]	Intercept	1	333.42	5394.78	**0.00**	3246.33	3254.65
	Time Window	3	336.96	1.11	0.34		
	Appraisal: Preparing family for your health changes	4	414.51	5.33	**0.00**		
	Appraisal * Time Window	12	359.44	1.37	0.18		
**Being independent and mobile**							
**Appraisal Main-Effect Model**	Intercept	1	195.72	8022.48	**0.00**	3313.29	3321.66
	Time Window	3	335.95	0.38	0.77		
	Appraisal: Being independent and mobile	4	446.50	5.11	**0.00**		
**Appraisal-by-time Interaction Model**	Intercept	1	204.98	7508.02	**0.00**	3248.19	3256.51
	Time Window	3	323.22	0.13	0.94		
	Appraisal: Being independent and mobile	4	418.61	3.63	**0.01**		
	Appraisal * Time Window	12	347.51	2.19	**0.01**		
**Shedding responsibilities**							
**Appraisal Main-Effect Model**	Intercept	1	241.72	6089.05	**0.00**	3307.16	3315.53
	Time Window	3	331.49	0.98	0.40		
	Appraisal: Shedding responsibilities	4	465.16	6.59	**0.00**		
**Appraisal-by-time Interaction Model**[ψ]	Intercept	1	248.15	5890.33	**0.00**	3252.71	3261.03
	Time Window	3	325.94	1.98	0.12		
	Appraisal: Shedding responsibilities	4	445.03	6.39	**0.00**		
	Appraisal * Time Window	12	353.20	1.01	0.44		

**Not complaining about your health**							
**Appraisal Main-Effect Model**	Intercept	1	204.96	7955.71	**0.00**	3299.06	3307.43
	Time Window	3	340.98	0.41	0.74		
	Appraisal: Not complaining about your health	4	448.30	8.83	**0.00**		
**Appraisal-by-time Interaction Model**	Intercept	1	209.63	7780.18	**0.00**	3233.26	3241.58
	Time Window	3	332.36	0.91	0.44		
	Appraisal: Not complaining about your health	4	420.81	8.36	**0.00**		
	Appraisal * Time Window	12	360.90	2.27	**0.01**		

Estimates of Fixed Effects: Dependent Variable: MCS Score

	Parameter		Mean Difference from Pre-Surgery	Std. Error	df	Sig.	95% Confidence Interval	
**Baseline Model**								
	Time Window							
		Pre-surgery	*referent*					
		6 weeks post-surgery	1.08	0.71	338.23	0.13	-0.31	2.47
		3 mo. post-surgery	1.26	0.74	341.79	0.09	-0.20	2.71
		12 mo. post-surgery	1.32	0.69	335.95	0.06	-0.04	2.68
**Positive outlook**								
**Appraisal Main-Effect Model**								
	Time Window							
		Pre-surgery	*referent*					
		6 weeks post-surgery	0.78	0.71	333.72	0.27	-0.61	2.17
		3 mo. post-surgery	1.23	0.74	342.92	0.10	-0.23	2.70
		12 mo. post-surgery	0.84	0.69	333.06	0.23	-0.52	2.21
	Appraisal: Positive Outlook							
		Never	*referent*					
		Rarely	-4.28	1.45	454.80	**0.00**	-7.13	-1.43
		Sometimes	-5.87	1.29	475.50	**0.00**	-8.41	-3.33
		Often	-5.31	1.15	480.18	**0.00**	-7.58	-3.05
		Always	-2.39	1.20	482.58	**0.05**	-4.74	-0.04

**Others' opinion**								
	**Appraisal Main-Effect Model**							
		Time Window						
		Pre-surgery	*referent*					
		6 weeks post-surgery	0.81	0.71	336.20	0.25	-0.58	2.21
		3 mo. post-surgery	0.58	0.74	344.83	0.43	-0.88	2.04
		12 mo. post-surgery	0.54	0.70	342.67	0.44	-0.84	1.91
		Appraisal: Others' Opinion						
		Never	*referent*					
		Rarely	-2.71	0.79	459.90	**0.00**	-4.27	-1.15
		Sometimes	-4.89	0.93	477.63	**0.00**	-6.72	-3.07
		Often	-5.93	1.13	484.89	**0.00**	-8.14	-3.71
		Always	-3.93	1.89	449.10	**0.04**	-7.63	-0.22
	**Appraisal*Time Window Interaction Model**							
		Time Window						
		Pre-surgery	*referent*					
		6 weeks post-surgery	1.27	0.82	346.05	0.12	-0.35	2.89
		3 mo. post-surgery	0.13	0.98	346.83	0.89	-1.80	2.07
		12 mo. post-surgery	4.38	1.41	339.63	**0.00**	1.60	7.16
		Appraisal: Others' Opinion						
		Never	*referent*					
		Rarely	-2.62	0.80	443.67	**0.00**	-4.18	-1.05
		Sometimes	-5.60	0.97	458.88	**0.00**	-7.50	-3.69
		Often	-5.20	1.17	469.55	**0.00**	-7.49	-2.90
		Always	0.26	2.43	363.32	0.92	-4.52	5.03
		Appraisal * Time Window Interaction						
		Pre-surgery * Rarely	-3.17	1.24	412.78	**0.01**	-5.60	-0.73
		Pre-surgery * Sometimes	-4.21	1.35	439.48	**0.00**	-6.86	-1.56
		Pre-surgery * Often	-8.39	1.53	452.66	**0.00**	-11.39	-5.38
		Pre-surgery * Always	-4.94	2.38	453.73	**0.04**	-9.62	-0.26
		6 weeks post-surgery * Rarely	-1.31	1.52	377.79	0.39	-4.29	1.68
		6 weeks post-surgery * Sometimes	-5.28	1.69	396.98	**0.00**	-8.60	-1.97
		6 weeks post-surgery * Often	-4.62	2.08	403.81	**0.03**	-8.71	-0.53
		6 weeks post-surgery * Always	c					
		3 mo. post-surgery * Rarely	-1.14	1.54	379.86	0.46	-4.16	1.88
		3 mo. post-surgery * Sometimes	-8.10	2.15	380.68	**0.00**	-12.32	-3.88
		3 mo. post-surgery * Often	-5.64	2.18	399.94	**0.01**	-9.92	-1.36
		3 mo. post-surgery * Always	-6.13	3.20	351.36	0.06	-12.43	0.16
		12 mo. post-surgery * Rarely	-4.86	1.45	389.41	**0.00**	-7.71	-2.00
		12 mo. post-surgery * Sometimes	-4.80	1.66	387.13	**0.00**	-8.06	-1.54
		12 mo. post-surgery * Often	-2.15	2.28	388.99	0.35	-6.63	2.34
		12 mo. post-surgery * Always	11.68	6.05	336.13	0.05	-0.23	23.58

**Money problems**								
**Appraisal Main-Effect Model**								
	Time Window							
		Pre-surgery	*referent*					
		6 weeks post-surgery	0.74	0.72	337.30	0.31	-0.69	2.16
		3 mo. post-surgery	1.11	0.74	338.05	0.14	-0.36	2.57
		12 mo. post-surgery	1.05	0.70	332.05	0.13	-0.32	2.42
	Appraisal: Money Problems							
		Never	*referent*					
		Rarely	-1.50	0.85	447.84	0.08	-3.17	0.17
		Sometimes	-4.29	0.99	484.50	**0.00**	-6.24	-2.34
		Often	-4.26	1.14	481.95	**0.00**	-6.50	-2.03
		Always	-3.07	1.40	483.21	**0.03**	-5.82	-0.31
**Preparing family for your health changes**								
**Appraisal Main-Effect Model**								
	Time Window							
		Pre-surgery	*referent*					
		6 weeks post-surgery	0.08	0.74	347.76	0.92	-1.38	1.54
		3 mo. post-surgery	0.01	0.79	357.94	0.99	-1.54	1.56
		12 mo. post-surgery	0.07	0.74	354.59	0.92	-1.38	1.52
	Appraisal: Preparing family for your health changes							
		Never	*referent*					
		Rarely	-2.29	0.85	452.17	**0.01**	-3.96	-0.63
		Sometimes	-2.76	0.96	466.65	**0.00**	-4.64	-0.89
		Often	-5.19	1.06	483.03	**0.00**	-7.28	-3.11
		Always	-5.01	1.50	465.93	**0.00**	-7.95	-2.07
**Being independent and mobile**								
**Appraisal Main-Effect Model**								
	Time Window							
		Pre-surgery	*referent*					
		6 weeks post-surgery	0.54	0.73	332.06	0.46	-0.90	1.98
		3 mo. post-surgery	0.62	0.77	340.57	0.42	-0.88	2.13
		12 mo. post-surgery	0.62	0.73	337.57	0.40	-0.81	2.05
	Appraisal: Being independent and mobile							
		Never	*referent*					
		Rarely	-2.37	1.12	422.36	**0.04**	-4.58	-0.17
		Sometimes	-1.63	1.02	442.37	0.11	-3.64	0.38
		Often	-3.78	1.02	472.08	**0.00**	-5.79	-1.76
		Always	-4.09	1.00	484.76	**0.00**	-6.05	-2.12

**Appraisal*Time Window Interaction Model**								
	Time Window							
		Pre-surgery	*referent*					
		6 weeks post-surgery	0.30	0.85	319.50	0.73	-1.38	1.98
		3 mo. post-surgery	0.43	0.78	328.17	0.58	-1.10	1.96
		12 mo. post-surgery	0.32	0.74	322.57	0.66	-1.13	1.77
	Appraisal: Being independent and mobile							
		Never	*referent*					
		Rarely	-2.54	1.19	386.50	**0.03**	-4.87	-0.21
		Sometimes	-2.02	1.03	414.33	0.05	-4.05	0.00
		Often	-3.52	1.04	452.55	**0.00**	-5.56	-1.48
		Always	-3.24	1.01	467.34	**0.00**	-5.24	-1.25
	Appraisal * Time Window Interaction							
		Pre-surgery * Rarely	-3.05	1.98	391.90	0.12	-6.95	0.85
		Pre-surgery * Sometimes	-1.11	1.72	413.51	0.52	-4.49	2.26
		Pre-surgery * Often	-5.23	1.65	413.50	**0.00**	-8.47	-1.98
		Pre-surgery * Always	-5.70	1.54	427.60	**0.00**	-8.73	-2.67
		6 weeks post-surgery * Rarely	-3.22	3.01	346.02	0.28	-9.13	2.69
		6 weeks post-surgery * Sometimes	-0.68	1.86	356.23	0.71	-4.33	2.96
		6 weeks post-surgery * Often	-0.57	1.86	379.10	0.76	-4.23	3.08
		6 weeks post-surgery * Always	-4.48	1.65	393.44	**0.01**	-7.71	-1.24
		3 mo. post-surgery * Rarely	-1.38	2.09	358.94	0.51	-5.50	2.73
		3 mo. post-surgery * Sometimes	-2.47	2.17	377.51	0.26	-6.74	1.80
		3 mo. post-surgery * Often	-4.37	2.00	369.76	**0.03**	-8.30	-0.44
		3 mo. post-surgery * Always	-4.23	1.89	378.41	**0.03**	-7.94	-0.52
		12 mo. post-surgery * Rarely	-2.52	1.81	357.31	0.16	-6.08	1.03
		12 mo. post-surgery * Sometimes	-3.83	1.98	367.91	0.05	-7.72	0.06
		12 mo. post-surgery * Often	-3.90	1.72	368.52	**0.02**	-7.28	-0.52
		12 mo. post-surgery * Always	1.43	1.81	379.85	0.43	-2.12	4.99
**Shedding responsibilities**								
**Appraisal Main-Effect Model**								
	Time Window							
		Pre-surgery	*referent*					
		6 weeks post-surgery	0.74	0.72	329.13	0.31	-0.68	2.16
		3 mo. post-surgery	1.05	0.76	332.19	0.17	-0.44	2.53
		12 mo. post-surgery	0.96	0.71	327.84	0.18	-0.43	2.35
	Appraisal: Shedding responsibilities							
		Never	*referent*					
		Rarely	-1.75	0.80	453.50	**0.03**	-3.32	-0.18
		Sometimes	-3.93	0.99	476.26	**0.00**	-5.88	-1.98
		Often	-5.02	1.17	484.48	**0.00**	-7.33	-2.72
		Always	-4.41	1.52	484.07	**0.00**	-7.40	-1.43
**Not complaining about your health**								
**Appraisal Main-Effect Model**								
	Time Window							
		Pre-surgery	*referent*					
		6 weeks post-surgery	0.60	0.72	336.68	0.41	-0.81	2.01
		3 mo. post-surgery	0.66	0.75	346.03	0.38	-0.82	2.14

		12 mo. post-surgery	0.58	0.71	341.51	0.42	-0.82	1.98
	Appraisal: Not complaining about your health							
		Never	*referent*					
		Rarely	-1.77	0.96	441.11	0.07	-3.66	0.12
		Sometimes	-2.79	0.96	461.24	**0.00**	-4.67	-0.91
		Often	-4.58	0.96	481.99	**0.00**	-6.47	-2.68
		Always	-5.81	1.09	480.15	**0.00**	-7.95	-3.67
**Appraisal*Time Window Interaction Model**								
	Time Window							
		Pre-surgery	*referent*					
		6 weeks post-surgery	1.00	0.74	330.07	0.18	-0.45	2.46
		3 mo. post-surgery	0.74	0.77	337.46	0.34	-0.77	2.24
		12 mo. post-surgery	0.94	0.74	328.38	0.20	-0.51	2.39
	Appraisal: Not complaining about your health							
		Never	*referent*					
		Rarely	-1.71	0.97	418.51	0.08	-3.62	0.20
		Sometimes	-3.11	0.97	432.59	**0.00**	-5.03	-1.20
		Often	-4.61	0.98	461.93	**0.00**	-6.54	-2.67
		Always	-5.49	1.09	462.07	**0.00**	-7.63	-3.35
	Appraisal * Time Window Interaction							
		Pre-surgery * Rarely	-2.84	1.55	409.33	0.07	-5.90	0.21
		Pre-surgery * Sometimes	-4.12	1.45	427.26	**0.00**	-6.97	-1.27
		Pre-surgery * Often	-5.67	1.40	438.55	**0.00**	-8.42	-2.92
		Pre-surgery * Always	-9.29	1.70	435.71	**0.00**	-12.62	-5.95
		6 weeks post-surgery * Rarely	0.89	1.93	364.02	0.64	-2.90	4.69
		6 weeks post-surgery * Sometimes	0.18	1.79	381.10	0.92	-3.34	3.71
		6 weeks post-surgery * Often	-5.20	1.77	389.45	**0.00**	-8.68	-1.73
		6 weeks post-surgery * Always	-6.13	2.00	406.76	**0.00**	-10.05	-2.20
		3 mo. post-surgery * Rarely	-2.68	2.08	385.29	0.20	-6.77	1.41
		3 mo. post-surgery * Sometimes	-2.86	1.84	371.54	0.12	-6.49	0.76
		3 mo. post-surgery * Often	-4.41	1.87	383.76	**0.02**	-8.10	-0.73
		3 mo. post-surgery * Always	-6.43	2.07	379.67	**0.00**	-10.51	-2.36
		12 mo. post-surgery * Rarely	-2.22	1.65	370.78	0.18	-5.46	1.03
		12 mo. post-surgery * Sometimes	-5.65	2.02	364.30	**0.01**	-9.62	-1.69
		12 mo. post-surgery * Often	-3.14	1.77	383.25	0.08	-6.62	0.33
		12 mo. post-surgery * Always	-0.12	1.89	388.07	0.95	-3.83	3.58

ᵠ Estimates of fixed effects not shown for this model because interaction was not statistically significant.

c. The level combination of factors in (I) is not observed.

Note: Bolded values have statistically significant p-values (p<0.05).
